# W-DARE: a three-year program of participatory action research to improve the sexual and reproductive health of women with disabilities in the Philippines

**DOI:** 10.1186/s12889-015-2308-y

**Published:** 2015-09-29

**Authors:** Cathy Vaughan, Jerome Zayas, Alexandra Devine, Liz Gill-Atkinson, Manjula Marella, Joy Garcia, Krissy Bisda, Joy Salgado, Carolyn Sobritchea, Tanya Edmonds, Sally Baker, Ma. Jesusa Marco

**Affiliations:** Centre for Health Equity, Melbourne School of Population and Global Health, The University of Melbourne, Victoria, 3010 Australia; Social Development Research Centre, De La Salle University, 2401 Taft Avenue, 1004 Manila, Philippines; Nossal Institute for Global Health, Melbourne School of Population and Global Health, The University of Melbourne, Victoria, 3010 Australia; WOWLEAP, 73 Edelweiss St., ESLA Urban Homes, Bgy. Sto. Domingo, Cainta, 1900 Rizal Philippines; PARE, CAPWD, Bgy. Calamba EcoCenter, Calamba, 6000 Cebu City, Philippines; Likhaan Center for Women’s Health, 27 St., Ofelia Subdivision, Ofelia, Project 8, Quezon City, 1106 Metro Manila Philippines; University of the Philippines Center for Women’s Studies Foundation, Magsaysay Ave., corner Ylanan St., Diliman, 1101 Quezon City, Philippines

**Keywords:** Disability, Sexual and reproductive health, Violence, Women, Participatory action research, Disability inclusive research, Mixed methods, Philippines

## Abstract

**Background:**

In many contexts, women with disability have less access to sexual and reproductive health information, screening, prevention, and care services than women without disability. Women with disability are also known to be more likely to experience physical and sexual violence than women without disability. In the Philippines, health service providers often have little awareness of the sexual and reproductive experiences of women with disability and limited capacity to provide services in response to their needs. Very limited data are available to inform development of disability-inclusive sexual and reproductive health, and violence prevention and response, services in the country. This paper presents the protocol for W-DARE (Women with Disability taking Action on REproductive and sexual health), a three-year program of participatory action research that aims to improve the sexual and reproductive health of women with disability in the Philippines.

**Design:**

W-DARE is a disability-inclusive program that will use mixed methods to 1) increase understanding of factors influencing the sexual and reproductive health of women with disability, and 2) develop, implement and evaluate local interventions to increase supply of and demand for services. W-DARE will generate data on the prevalence of disability in two districts; the wellbeing and community participation of people with and without disability, and identify barriers to community; and describe the sexual and reproductive health needs and experiences, and service-related experiences of women with disability. These data will inform the development and evaluation of interventions aiming to improve access to sexual and reproductive health services, and violence prevention and response services, for women with disability. Local women with disabilities, their representative organisations, and SRH service providers will be involved as members of the research team across all stages of the research.

**Discussion:**

This three-year study will provide evidence about factors undermining the sexual and reproductive health of women with disability in a lower-middle income country, and provide new insights about what may be effective in increasing access to services in settings of limited resources. Findings will be relevant across Asia and the Pacific. Analysis of the program will also provide evidence about disability-inclusion in participatory action research approaches.

## Background

An estimated 15 percent of the world's population, or 1 billion people, live with disability [1]. Women with disability have greater unmet health needs than women without disability, and reduced access to health information, screening, prevention, and care services [2, 3]. In particular, women with disability have minimal access to sexual and reproductive health (SRH) programs [2]. They have often been excluded from development activities promoting access to SRH information and services because having a disability is falsely associated with being asexual [4]; because SRH services are often not appropriate for, or accessible to, women with disability [5, 6]; and because there are limited accurate data on the prevalence of disability, and on the SRH experiences and needs of women with disability available to inform disability inclusive SRH activities [2].

A range of factors undermines the SRH of women with disability. These include direct factors (environmental barriers, service costs, lack of accessible transportation, lack of services, care-givers assuming women with disability do not need SRH information or services) and in-direct factors (lack of education and access to economic resources, limited agency, lack of awareness of rights) [7]. The limited economic participation and independence of women with disability reduces their ability to access basic services of all kinds, including SRH services [7]. Across the world, women with disability face challenges to their right to SRH information; prejudice and discrimination from service providers; and barriers associated with local attitudes towards disability, women and SRH [2, 5, 7]. In addition, women with disability are considerably more likely to be victims of physical and sexual abuse than women without disability [8, 9], further compromising their health overall and SRH specifically [10, 11].

Article 32 of the UN Convention on the Rights of Persons with Disabilities (CRPD) emphasises the importance of including people with disability in development. The CRPD also has specific provisions related to recognition of reproductive rights (Article 23); access of people with disabilities to SRH information and services (Article 25); and the rights and empowerment of women (Article 6) [12]. For the freedoms set out in the CRPD to be achieved, women with disability need to be provided with age appropriate, accessible information on SRH, and have their rights to be involved in a sexual relationship, marry, establish a family, enjoy reproductive health, and to physical integrity recognised [9]. However efforts to uphold elements of the CRPD throughout Asia and the Pacific are hampered by the lack of context specific data about the prevalence of disability and about women’s experiences in relation to SRH and protection from violence, including women’s expressed needs, priorities and perceptions of services. In addition, there is very limited information available as to ‘what works’ to address barriers and increase access to SRH and protection from violence information and services, for women with disability.

### The context for women with disability in the Philippines

Based on the 2010 census, the Philippines Statistics Authority estimate that 1.57 % of the population over the age of five has a disability and that just under half of all people with disability are women [13]. However, it is thought that the real number of people with disability in the Philippines might actually be much higher due to the methods used to collect disability data and underreporting arising from pervasive cultural misconceptions and prejudices about disability [14].

It is known that at least one in five women in the Philippines, aged 15 – 49, experience physical violence [15]. Though national data on women’s experience of violence are not disaggregated by disability, evidence from other countries in the region suggests the rate will be higher for women with disability [9, 11]. SRH and other issues important to women with disability in the Philippines are largely under-researched, but those studies that have been conducted highlight that women with disability, especially those with low and middle socio-economic status, are more likely to experience human rights violations than any other demographic group in the Philippines [16, 17]. In 2012, UNFPA supported a situation analysis that identified an urgent need for further research to inform the development of disability inclusive health service policy and programming by relevant government agencies in the Philippines [16].

W-DARE (Women with Disability taking Action on REproductive and sexual health) is a program of participatory action research designed to address this research gap, and aims to contribute to the evidence available to policy makers in the Philippines and in other lower- and middle-income countries. This paper presents the protocol for W-DARE (referred to originally in funding documents as *Sexual and Reproductive Health of Women with Disability in the Philippines: Building evidence for action*) outlining our participatory action approach and the research methods used during different phases of the three-year program.

W-DARE is being led by researchers from the University of Melbourne (Australia) and De La Salle University (Philippines), in partnership with national Disabled People’s Organisations (DPOs) WOWLEAP and PARE; the non-profit SRH service provider Likhaan Center for Women’s Health; and the Center for Women’s Studies Foundation (University of the Philippines). The W-DARE team includes formally trained researchers (academics), disability inclusive development specialists, SRH service providers and women with disability.

### Aims of W-DARE

The overall aim of W-DARE is to improve access to SRH services and information, including protection from violence, for women with disability in the Philippines. The specific objectives of the research are to:

1. Increase the availability of information on disability prevalence and the lived experience of people with disability in the Philippines.
2. Generate data on the experiences of women with disability in relation to their SRH, SRH needs, access to SRH programs, and experiences of SRH programs in Quezon City (Metro Manila) and Ligao City (Albay Province).
3. Design and trial interventions aiming to increase access to SRH programs for women with disability in the two research sites.
4. Assess the effectiveness of these interventions in order to develop guidelines for gender-sensitive disability inclusion in SRH programs and across the health sector in the Philippines.
5. Increase the capacity of local partners to implement disability inclusive research.

### Theoretical framework

The approach to disability used in W-DARE is informed by the CRPD, which notes *“Disability results fromthe interaction between persons with impairments and attitudinal and environmental barriers that hinders their full and effective participation in society on an equal basis with others”* [12]. This reflects that it is not only an individual’s impairment that limits or prevents their participation in society, including access to SRH. The social model of disability [18], which emphasises the impact of physical, attitudinal and other barriers to SRH, and a human rights based approach to disability [19] inform our approach to analysis of data and design of interventions. However, we recognise the impact of impairment as important in the lives of many women with disability and that a barrier-free society is an aspirational rather than achievable goal [20], particularly in the context of a lower-middle income country. Therefore we take a pragmatic approach, being guided by the priorities and strategies of women with disability who are partners throughout this program of participatory action research.

Since the early 1970s participatory action research has increasingly been used in a wide range of settings, but particularly in contexts of poverty, inequality and exclusion [21]. Participatory action research practices recognise that research methods should support overall aims of empowerment and social justice and aim to shift the alignment of power within the research process through the participation of research beneficiaries and subjects [22]. In recent years, participatory action research approaches have become widely accepted in disability research as they aim to appropriately access and represent the views and experiences of people with disability, and reflect that people with disability need to be treated with respect by the research community [22]. It is important to the W-DARE partners that the research increases knowledge about the barriers to SRH for women with disability in the Philippines, from women’s own perspective. The research team also believe that it is critical to respond to the question of ‘what to do about it’, if W-DARE is going to make a difference to the SRH of women with disability. As such, we have designed a program of participatory action research that includes the implementation and evaluation of interventions.

## Methods/Design

W-DARE is a mixed method disability-inclusive program of participatory action research that will be conducted from April 2013 - March 2016. The three-year program of research involves generation and analysis of information about disability in the Philippines, and specifically about the SRH experiences and needs of women with disability; development and implementation of interventions to address identified barriers to SRH; evaluation of the efficacy of these interventions; and engagement with national stakeholders to inform local replication and adaptation in other parts of the country. Research findings will inform the development of guidelines for gender-sensitive disability inclusion in SRH programs and across the health sector in the Philippines. Methods include a cross-sectional population-based household survey, in-depth interviews, focus group discussions, pre- and post-intervention questionnaires, and collection of stories of change. Consistent with a participatory action research approach, those most affected by the limited access to SRH programs for women with disability (that is, women with disability and SRH service providers) will be involved in all phases of the research including initial design, development of research tools and processes, collection and analysis of data, design and implementation of interventions, and drafting of guidelines.

### Study locations

W-DARE is being conducted in the Local Government Units of Quezon City in Metro Manila and Ligao City in Albay Province. Quezon City (QC) is the most populous city in the Philippines and therefore W-DARE is focusing primarily on one district of the city, District 2. District 2 has an estimated population of 635,967 [23] with households from both extremes of the socio-economic spectrum, but overall is relatively disadvantaged with many informal settler families living in the district. Ligao City (LC) is a rural and semi-urban district in Albay Province, approximately one hour from the Provincial Capital Legazpi. Running from the foot of Mt. Mayon to the coast in the southwest, the district has a population of 104,914 [23]. These locations were selected to provide data on the experience of women from across the socio-economic spectrum and living in diverse settings including households in rural communities, and those in the highly urbanised cities found throughout Philippines, including in informal settlements.

### Training and capacity building

The research design includes a range of capacity development activities to ensure that research partner organisations and representatives have the skills and confidence to be actively involved throughout W-DARE. Training workshops aim to improve the capacity of project partners to conduct disability-inclusive research using quantitative and qualitative methods, and to ensure that all individuals and partner organisations have a shared understanding of the aims and objectives of the research as well as the ethical considerations associated with data collection, analysis and dissemination. Workshops also aim to increase the ability of external researchers to understand the Philippines context, to engage with local priorities and sensitivities, and to adapt processes to ensure they are appropriate for the Philippines. Individual workshops will be conducted in each research site, as well as trainings that bring representatives from both research sites together.

### Data collection – household survey

The aim of the household survey is to estimate prevalence of disability among the population aged 18 years and above in District 2 of QC and in LC. The household survey will also enable investigation of socio-demographic factors associated with disability; comparison of well-being and access to community between people with and without disability; and comparison of SRH knowledge and access to SRH services between women with and without disability.

### Tools

In order to establish disability prevalence in the two research sites W-DARE will use the *Rapid Assessment of Disability (RAD) Questionnaire* developed by the Nossal Institute for Global Health and the Centre for Eye Research Australia at the University of Melbourne [24]. The RAD is a population-based household survey designed to identify people with disability, and measure well-being and access to community for people with disability. The *RAD* questionnaire is interviewer administered and has two parts: the first part contains questions about the socio-economic characteristics of the household, which are administered to the head of the household; the second part is a questionnaire administered to each individual in the household and contains sections on demographic information, self-assessment of functioning, well-being and access to the community. The second section of the *RAD* uses questions on self-reported functional difficulty across domains similar to those used by the Washington Group on Disability Statistics as a measure of disability [25]. These include questions about vision, hearing, communication and mobility; about gross motor, fine motor, cognitive and psycho-social functioning; and about self-reported difficulty associated with appearance and psychological wellbeing. The questionnaire also has sections designed to capture aspects of wellbeing and access to the community for respondents who report functional difficulty and their age (with an accepted difference of two years) and gender matched controls (people without disability) from neighbouring households, to allow for comparisons between people with and without disability. The *RAD* will be conducted in District 2 of QC and in LC.

An additional *Women’s Health Questionnaire*, to measure women’s access to SRH services and information, will be administered to all women identified as having functional limitation using the *RAD* and to matched controls. Inclusion of matched controls will enable the team to compare access to SRH services for women with and without disability. Development of the *Women’s Health Questionnaire* was informed by existing tools including the Philippines census questions, DHS surveys, and surveys developed by international agencies such as WHO and UNFPA. Cognitive testing of the *RAD* and *Women’s Health Questionnaires* will be conducted in both QC and LC, including with respondents with a range of impairments.

Questionnaires will be translated in to Tagalog and then back translated in to English. They will also be cognitively tested on a convenience sample with different disabilities to ensure a range of respondents understand the questions as intended and that their responses accurately reflect what is being asked. Permission to conduct the household survey will be sought from barangay *kapitans* at each research site, and fieldwork teams will be accompanied by local barangay health workers. Fieldwork teams will include women and men with disability and representatives of project partner organisations, and will be supported and supervised by experienced co-investigators from De La Salle University and the University of the Philippines.

### Sample and recruitment

A sample size of 3010 for the household survey has been estimated using a disability prevalence of 5 % [13, 26] a 95 % confidence level, sampling error of 20 %, an estimated design effect of 1.5, and a non-response rate of 10 %. Assuming recruitment of 50 people from each cluster, this means a total of 60 clusters are required to complete the household survey.

The cross-sectional population-based survey will use two-staged cluster random sampling. In the first stage of sampling, groups of puroks in QC and barangays in LC (‘clusters’) will be randomly selected with probability-proportionate to size. Each cluster will be mapped based on information from the National Statistics Office, and divided into equal segments of houses estimated to provide 50 potential participants aged 18 years and above.

One segment from each cluster will be randomly selected for data collection. Within the selected segment, the survey team will visit all households door-to-door accompanied by the local barangay health worker. The head of household will be informed about the study and invited to complete the first part of the *RAD* (about household socio-economic characteristics). All individuals living in the household and aged 18 years and above will be provided with translated information about the project in the form of a plain language statement and invited to complete the individual questionnaire. Interested persons will be asked to provide informed consent (written or verbal, as appropriate). If an eligible household member is absent, at least two return visits will be made to the household in an attempt to minimise recruitment bias associated with the timing of data collection. The survey teams have access to sign language interpreters to facilitate recruitment of any Deaf potential participants. Door-to-door visits will continue in each segment until 50 people aged 18 years and above have been recruited. Women identified as having functional limitation through the *RAD* will be asked to also complete the *Women’s Health Questionnaire,* as will matched controls (women without disability from approximately the same location and age group).

### Data collection–in-depth interviews and focus group discussions

Qualitative data will also be collected using in-depth interviews and focus group discussions to increase understanding of the SRH needs and experiences, SRH service-related experiences and priorities of women with disability in QC and LC, from the perspectives of the women themselves, their families and partners, service providers and policy makers. Question guides will be designed to explore barriers to SRH and violence services, as well as factors that increase access and uptake by women with disability.

Approximately 25 interviews (18 in QC and 7 in LC) will be conducted with women with disability and 10 interviews (7 in QC and 3 in LC) with girls with disability aged 15 to 17 years; 15 interviews (10 in QC and 5 in LC) will be conducted with SRH service providers; and 9 focus group discussions (5 in QC and 4 in LC, with a total of approximately 70 participants) will be conducted with partners and families of women with disability, and with women without disability. Recruitment of participants will continue until theoretical saturation is achieved within the data.

Women with disability, their partners and families will be identified through the membership and networks of partner DPOs and non-government organisations active in QC and/or LC. Women identified as having a functional impairment during the implementation of the RAD, and their partners and carers, will also be invited to participate in the qualitative data collection activities. All participants will be 18 years of age or above. All participants will be provided with a plain language statement, and will be asked to give either written or verbal consent. Interviews and focus groups will be conducted in Tagalog and/or English, and audio-recorded with participant permission.

Qualitative data collectors will include experienced Filipina academic researchers and women with disability with previous experience conducting interviews. All data collectors will participate in a range of capacity building activities, including training in disability and gender inclusive research, a workshop to develop jointly agreed question guides, training in the use of qualitative data collection tools (including training in referral and reporting protocols), and a participatory data analysis workshop to interrogate early thematic analysis of the data.

### Analysis of household data

Household survey data will be entered locally in the Philippines in a custom built Access database to ensure high data quality by restricting data that can be entered. Asset index will be used as a proxy indicator for wealth status using principal components analysis on the data from the household questionnaire [27]. Households will then be divided into quintiles with the first quintile representing the poorest in the sample, and the fifth quintile representing the wealthiest. Chi square and Fisher’s exact test (used when any expected frequency is less than 5) will be performed to determine whether disability is associated with demographic and socio-economic characteristics. Multivariate logistic regression models will be performed to assess the presence of statistically significant associations between socio-economic risk factors and prevalence of disability as measured by the *RAD*. Confidence intervals for prevalence estimates and regression odds ratios will be calculated with adjustment for clustering effects in the study design using the generalised estimating equation approach. Age and gender adjusted prevalence rates will be derived using projected population estimates for 2014 as the reference standard.

### Analysis of qualitative data

Data collected during in-depth interviews and focus group discussions will be translated (from Tagalog into English) where necessary, and transcribed verbatim. Early deductive analysis of data will be done collaboratively with partner agencies and the individuals that were involved in data collection through a participatory analysis workshop, during which draft data coding frameworks will be developed. Data will then be entered into the qualitative analysis software QSR NVivo for further inductive data-driven coding of text and thematic analysis.

### Planning, prioritising and assessing effectiveness of interventions

Quantitative and qualitative data will be analysed to identify key barriers to SRH for women with disability, as well as facilitators of SRH that can be built on. W-DARE researchers will conduct participatory workshops with members of the data collection team, partner organisations and local stakeholders to identify possible interventions to address these barriers and maximise facilitators. Potential interventions will be prioritised on the basis of a collective assessment of need, likely impact, feasibility, support from policy makers and power brokers, and the resources available to the program.

Interventions are anticipated to include activities to increase the supply of SRH services and information to women with disability, and activities to increase demand for SRH services and information. Evaluation of the effectiveness of interventions will use mixed methods and be based on analysis of quantitative data (with potential data including review of the number of women with disability accessing SRH and violence services; review of pre- and post-training data; and analysis of data about wellbeing and access to community collected using the RAD before and after interventions with women with disability); qualitative data (with data potentially being generated through follow up interviews with service providers and women with disability some months after the completion of interventions; collection of stories of change with participants in interventions); and operational data (including, for example, facility accessibility audits; review of facility or local government budgetary commitments to disability inclusion). Data collection processes will be finalised during the development and implementation of activities, consistent with the participatory action research approach of the overall program.

### Ethical and safety issues

Participants who discuss their personal experiences in relation to SRH (including exposure to violence) may find this distressing. Even when capable of giving consent, women with intellectual disability, psychosocial disability and/or communication impairments, may be more than usually vulnerable to discomfort or stress arising from participating in the research.

A comprehensive safety protocol has been developed for the project, to minimise risks for participants and for members of the research team. This protocol includes guidance on responding to disclosure of violence against women with disability; referral pathways and supports to ensure that participants can access local services; and on strategies for adapting research processes to be inclusive of women with different types of disability including intellectual and psychosocial disability.

The study will be conducted in accordance with the tenets of the Declaration of Helsinki. All participants will be informed about the study using a plain language statement in Tagalog and interested participants will be asked to provide written or verbal informed consent to participate and for publication of findings noting that they will not be individually identified. For participants who are not literate, the consent form will be read to them and their verbal agreement will be recorded by the interviewer in front of a witness. Parents or guardians of girls with disability aged 15 to 17 years will be asked for their consent, in addition to girls themselves. The University of Melbourne Human Research Ethics Committee in Australia (ethics ID 1339640) and the De La Salle University Ethics Committee in the Philippines have granted W-DARE ethics approval.

## Discussion

W-DARE will increase the availability of information on disability prevalence and the lived experience of disability, and generate data on the experiences of women with disability in relation to SRH and access to SRH programs. Findings will be generated from an urban and a rural district in the Philippines, but are anticipated to be relevant to other settings across the country and in other lower-middle income countries. The W-DARE program will evaluate interventions to increase access to SRH programs for women with disability, providing evidence in relation to the crucial question of what can be done to improve SRH and address barriers for women with disability. Analysis of the participatory approaches used throughout the program will also increase understanding of the processes and impacts of disability inclusive research.

Few study protocols for mixed method programs of participatory action research have been published. This is especially true in relation to research with women with disabilities in settings of limited resources. We hope that by outlining the disability-inclusive, participatory action research processes used in the W-DARE program we can contribute to addressing this knowledge gap, and encourage increased use of participatory research approaches by researchers working to increase disability-inclusion in development.

## Abbreviations

CRPD: United Nations Convention on the Rights of Persons with Disabilities
DPO: Disabled People’s Organisation
LC: Ligao City
PARE: Persons with disability Advocating for Rights and Empowerment
QC: Quezon City
RAD: Rapid Assessment of Disability questionnaire
SRH: Sexual and Reproductive Health
UNFPA: United Nations Population Fund
W-DARE: Women with Disability taking Action on REproductive and sexual health
WOWLEAP: Women with disability leap social and economic progress
